# Supplementary material for: Methodological issues in visible LED therapy dermatological research and reporting
Source: PLoS One. 2025 Oct 1;20(9):e0332995. doi: 10.1371/journal.pone.0332995 (PMC12488025; doi:10.1371/journal.pone.0332995)
Supplement: Attachment — Submitted filename: Reply to reviewers.docx [file pone.0332995.s002.docx]

Reply to reviewers

3^rd^ September 2025

[Davidrobert.grimes@tcd.ie](mailto:Davidrobert.grimes@tcd.ie)

**Re: PONE-D-25-29256: Methodological issues in visible LED therapy dermatological research and reporting**

Dear Prof Hatahet and reviewer,

Thank you very much for your constructive feedback on this manuscript above, and your time spent on it. I will endeavour to reply to both editorial and reviewer comments in this document, please don’t hesitate to contact me if I can provide anything further!

Best wishes and thanks again,

David

# Editorial responses

1. Please ensure that your manuscript meets PLOS ONE's style requirements, including those for file naming.

I believe this is the case now, please advise if changes required.

2. Thank you for stating the following financial disclosure: [This work has been funded by the Wellcome Trust (Grant number 214461/A/18/Z).]. Please state what role the funders took in the study. If the funders had no role, please state: "The funders had no role in study design, data collection and analysis, decision to publish, or preparation of the manuscript." If this statement is not correct you must amend it as needed. Please include this amended Role of Funder statement in your cover letter; we will change the online submission form on your behalf.

Thank you, the statement should be: “This work has been funded by the Wellcome Trust (Grant number 214461/A/18/Z). The funders had no role in study design, data collection and analysis, decision to publish, or preparation of the manuscript”

3. Thank you for stating the following in your manuscript: [This work has been funded by the Wellcome Trust (Grant number 214461/A/18/Z).] We note that you have provided funding information that is currently declared in your Funding Statement. However, funding information should not appear in the Acknowledgments section or other areas of your manuscript. We will only publish funding information present in the Funding Statement section of the online submission form. Please remove any funding-related text from the manuscript and let us know how you would like to update your Funding Statement. Currently, your Funding Statement reads as follows

This has now been deleted. Please see reply to point 3 above.

4. In the online submission form, you indicated that [Code to run all the analysis in this work is hosted on GitHub ( https://github.com/drg85/LEDcheck ) and further analytical software in various languages is available by request from the author.]. All PLOS journals now require all data underlying the findings described in their manuscript to be freely available to other researchers, either 1. In a public repository, 2. Within the manuscript itself, or 3. Uploaded as supplementary information

The code is all available on the github link supplied, and the data (solar spectra) publicly available to all researchers. I have included a copy on github, but it is the American Society for Testing and Materials (ASTM) G-173 spectra for terrestrial solar spectral irradiance which is already a public resource.

# Reviewer responses

1. It is interesting paper and important especially at academic as well as social point of view. As the author mentions, standardization of light characteristic is mandatory to evaluate impacts of therapeutic modalities with light (LED of course or even UV light) on exposed human body. Without knowing this, we, not only non-professional uses but also medical professional, might just feel like we understand it. Particular interest should be focused on comparison with hours of equivalent solar exposure time. This may objectify fluence of each LED tools. Only question to the author is difference of non-Gaussian- from Gaussian-LED light spectrum when compared with equivalent solar exposure. I would think that comparison with non-Gaussian light spectrum might be much difficult and complicated than Gaussian ones. Of course, as a matter of fact, I wouldn't think that non-Gaussian LED spectrum is superior, however, it is better stated in the discussion section when such devices are available in the market presently.

This is a very astute point. I’ve added some text in the discussion to address this primary concern raised, and also to consider the possibility of future filtered white light LEDs etc: The first next text is;

“This work also has limitations that need to be elucidated. Firstly, while the equivalent solar exposure time will have the same fluence and central wavelength as a given quasi-monochromatic LED, the non-Gaussian nature of solar spectra means the precise wavelength distribution will be a function of the solar irradiance spectra. Critically, tThe solar equivalent dose calculation also pivots on the fundamental assumption that the LED spectra is approximately Gaussian. This is however a reasonable assumption, and most LEDs are sold with their FWHM quoted on this basis”.

And the second addition:

“Another potential limitation is that the work outlined assumes all devices are centred around a particular wavelength in red, blue, or yellow, which is determined by the LED type. This does indeed appear to be the case, but it is also possible that a hypothetical device could use a white LED and then a colour filter, in which case the line shape will be influenced by the combination of the white LED emission and the filter transmission. This could be markedly different from the Gaussian profile assumptions elucidated here, as the emission from white LEDs is due to phosphors excited by the emission from a ultraviolet LED at the centre of the structure, a physical emission process entirely different to an electrically injected LED with emission determined solely by semiconductor bandgap. White LEDs are however considerably more expensive than quasi-monochromatic LEDs, as the requirement for a phosphor coating drives up prices, and also have much lower efficiency. For this reason, devices for which specifications were available simply used quasi-monochromatic LEDs for PMB. In these cases, the analysis presented here holds, but the paucity of reporting of the technical specifications of many of these devices can make this hard to determine. “
